# Chronic Obstructive Pulmonary Disease in Elderly Patients with Acute and Advanced Heart Failure: Palliative Care Needs—Analysis of the EPICTER Study

**DOI:** 10.3390/jcm11133709

**Published:** 2022-06-27

**Authors:** Manuel Méndez-Bailón, Noel Lorenzo-Villalba, Miriam Romero-Correa, Claudia Josa-Laorden, Luis Inglada-Galiana, Eva Menor-Campos, Noelia Gómez-Aguirre, Carolina Clemente-Sarasa, Rosario Salas-Campos, Carmen García-Redecillas, María Asenjo-Martínez, Joan Carles Trullàs, Begoña Cortés-Rodríguez, Carla de la Guerra-Acebal, Ana Serrado Iglesias, Reyes Aparicio-Santos, Francesc Formiga, Emmanuel Andrès, Oscar Aramburu-Bodas, Prado Salamanca-Bautista, on behalf of EPICTER Study Group

**Affiliations:** 1 Servicio de Medicina Interna, Hospital Clínico Universitario San Carlos, Universidad Complutense de Madrid Instituto de Investigación Sanitaria (IdISSC), 28040 Madrid, Spain; manuelmenba@hotmail.com; 2 Service de Médecine Interne, Diabète et Maladies Métaboliques, Hôpitaux Universitaires de Strasbourg, 67000 Strasbourg, France; 3 Servicio de Medicina Interna, Hospital General de Riotinto, 21660 Huelva, Spain; mirimedd@hotmail.com (M.R.-C.); emmanuel.andres@chru-strasbourg.fr (E.A.); 4 Servicio de Medicina Interna, Hospital Clínico Universitario Lozano Blesa, 50009 Zaragoza, Spain; claudiajosa@gmail.com; 5 Servicio de Medicina Interna, Hospital Universitario Río Hortega, 47012 Valladolid, Spain; ingladagaliana0@gmail.com; 6 Servicio de Medicina Interna, Hospital Universitario de Jerez de la Frontera, 11407 Jerez de la Frontera, Spain; evamenor@gmail.com; 7 Servicio de Medicina Interna, Hospital Ernest Lluch Martín, 50299 Calatayud, Spain; nga2006@hotmail.es; 8 Servicio de Medicina Interna, Hospital Royo Villanova, 50015 Zaragoza, Spain; carolina_clemente8@hotmail.com; 9 Servicio de Medicina Interna, Hospital Universitario Sagrat Cor, 08029 Barcelona, Spain; rsalascampos@yahoo.es; 10 Servicio de Medicina Interna, Complejo Hospitalario de Jaén, 23007 Jaén, Spain; garciaredecillas@gmail.com; 11 Servicio de Medicina Interna, Hospital Universitario Rey Juan Carlos, 28933 Móstoles, Spain; mariaasenjomartinez@gmail.com; 12 Servicio de Medicina Interna, Hospital d’Olot i Comarcal de la Garrotxa, 17800 Olot, Spain; jctv5153@comg.cat; 13 Servicio de Medicina Interna, Hospital Alto Guadalquivir, 23740 Andújar, Spain; bcortes@ephag.es; 14 Servicio de Medicina Interna, Hospital de Mendaro, 20850 Mendaro, Spain; carla.dlga@gmail.com; 15 Servicio de Medicina Interna, Hospital Municipal de Badalona, 08911 Badalona, Spain; aserrado@bsa.cat; 16 Servicio de Medicina Interna, Hospital San Juan de Dios del Aljarafe, 41930 Bormujos, Spain; reyesapa@yahoo.es; 17 Servicio de Medicina Interna, Hospital Universitario de Bellvitge, 08907 Barcelona, Spain; fformiga@bellvitgehospital.cat; 18 Servicio de Medicina Interna, Hospital Universitario Virgen Macarena, 41009 Sevilla, Spain; oscarab2000@gmail.com (O.A.-B.); pradosalamanca@gmail.com (P.S.-B.); 19 Department of Medecine, Universidad de Sevilla, San Fernando, 4, 41004 Sevilla, Spain

**Keywords:** hronic obstructive pulmonary disease, advance heart failure, palliative care

## Abstract

**Introduction:** There are studies that evaluate the association between chronic obstructive pulmonary disease (COPD) and heart failure (HF) but there is little evidence regarding the prognosis of this comorbidity in older patients admitted for acute HF. In addition, little attention has been given to the extracardiac and extrapulmonary symptoms presented by patients with HF and COPD in more advanced stages. The aim of this study was to evaluate the prognostic impact of COPD on mortality in elderly patients with acute and advanced HF and the clinical manifestations and management from a palliative point of view. **Methods:** The EPICTER study (“Epidemiological survey of advanced heart failure”) is a cross-sectional, multicenter project that consecutively collected patients admitted for HF in 74 Spanish hospitals. Demographic, clinical, treatment, organ-dependent terminal criteria (NYHA III-IV, LVEF <20%, intractable angina, HF despite optimal treatment), and general terminal criteria (estimated survival <6 months, patient/family acceptance of palliative approach, and one of the following: evidence of HF progression, multiple Emergency Room visits or admissions in the last six months, 10% weight loss in the last six months, and functional impairment) were collected. Terminal HF was considered if the patient met at least one organ-dependent criterion and all the general criteria. Both groups (HF with COPD and without COPD) were compared. A Kaplan–Meier survival analysis was performed to evaluate the presence of COPD on the vital prognosis of patients with HF. **Results:** A total of 3100 patients were included of which 812 had COPD. In the COPD group, dyspnea and anxiety were more frequently observed (86.2% vs. 75.3%, *p* = 0.001 and 35.4% vs. 31.2%, *p* = 0.043, respectively). In patients with a history of COPD, presentation of HF was in the form of acute pulmonary edema (21% vs. 14.4% in patients without COPD, *p* = 0.0001). Patients with COPD more frequently suffered from advanced HF (28.9% vs. 19.4%; *p* < 0.001). Consultation with the hospital palliative care service during admission was more frequent when patients with HF presented with associated COPD (94% vs. 6.8%; *p* = 0.036). In-hospital and six-month follow-up mortality was 36.5% in patients with COPD vs. 30.7% in patients without COPD, *p* = 0.005. The mean number of hospital admissions during follow-up was higher in patients with HF and COPD than in those with isolated HF (0.63 ± 0.98 vs. 0.51 ± 0.84; *p* < 0.002). Survival analysis showed that patients with a history of COPD had fewer survival days during follow-up than those without COPD (log Rank chi-squared 4.895 and *p* = 0.027). **Conclusions:** patients with HF and COPD had more severe symptoms (dyspnea and anxiety) and also a worse prognosis than patients without COPD. However, the prognosis of patients admitted to our setting is poor and many patients with HF and COPD may not receive the assessment and palliative care support they need. Palliative care is necessary in chronic non-oncologic diseases, especially in multipathologic and symptom-intensive patients. This is a clinical care aspect to be improved and evaluated in future research studies.

## 1. Introduction

The incidence and prevalence of heart failure (HF) and chronic obstructive pulmonary disease (COPD) is continuously increasing. HF prevalence has been reported to be higher in patients with COPD compared to the general population (10–30% versus 1–2%) [1,2]. HF is a common condition in patients hospitalized for exacerbation of COPD: in these patients, 20% have been described to have pre-existing HF while 40% have new HF [3]. Patients presenting both comorbid conditions (HF and COPD) are likely to be smokers and older with an important burden of comorbid conditions compared to patients with HF without COPD [1,3,4].

Clinically, the association of these two conditions is particularly challenging as they can present with similar clinical manifestations, which can significantly delay the diagnosis of HF [5,6]. In addition, HF remains underdiagnosed and undertreated in patients with COPD, with a one year mortality rate reaching 50% [3,7]. Thus, COPD is associated with increased cardiovascular morbidity and mortality independent of the left ventricular ejection fraction (LVEF) [8].

HF with preserved or reduced LVEF should not be treated differently if COPD coexists (1) and HF guidelines should be followed. However, beta-blockers are underprescribed in these patients due to concerns regarding their side effects on pulmonary function [1,9]. In a large retrospective analysis from an HF registry, beta-blocker selectivity was not associated with a difference in outcomes for patients with HF with COPD as compared with those with HF without COPD [10]. In addition, benefits from beta-blockers outweigh potential risks even in patients with severe COPD [1,10]. Regarding the impact of treatment with sacubitril/valsartan on pulmonary function, no data are available.

There are studies that evaluate the association between COPD and HF but there is little evidence regarding the prognosis of this comorbidity in older patients admitted for acute HF. In addition, little attention has been given to the extracardiac and extrapulmonary symptoms presented by patients with HF and COPD in more advanced stages.

The aim of this study was to evaluate the prognostic association of COPD on mortality in elderly patients with acute HF and to evaluate the clinical manifestations and management from a palliative point of view.

## 2. Material and Methods

### 2.1. Study Population

The EPICTER study (“Epidemiological survey of advanced heart failure”) is a cross-sectional and prospective, multicenter project that consecutively collected data on patients admitted for HF in 74 Spanish public or private hospitals, regardless of hospital size. Patients were recruited in two periods (summer and winter). To avoid bias, hospitals began collecting data on the same day (1 June and 30 November 2016) in which all patients admitted to Cardiology or Internal Medicine departments, Intensive Care Units, or any other service were included. Researchers at each center checked patients who met the inclusion criteria daily and each hospital continued to recruit patients on subsequent days until the required number was reached. The minimum number of patients to be included for each hospital was pre-determined according to the number of hospital beds. Inclusion criteria were (1) age older than 18 years, (2) admission to the hospital room before 8:00 o’clock on the day of data collection, (3) HF as the main cause of admission: acute HF, acute pulmonary edema, acute coronary syndrome Killip III-IV, or cardiogenic shock. Exclusion criteria were (1) patients attended in the Emergency Department, but not yet admitted, and (2) patients who did not sign the informed consent. All patients received the usual treatments and medical care and were classified into two groups according to whether or not they had no COPD.

### 2.2. Study Variables

Demographic, clinical, treatment, organ-dependent terminal criteria (NYHA III-IV, LVEF < 20%, intractable angina, HF despite optimal treatment), and general terminal criteria (estimated survival <6 months, patient/family acceptance of palliative approach, and one of these: evidence of HF progression, multiple ER visits or admissions in the last 6 months, 10% weight loss in the last 6 months, functional impairment) were collected. Terminal HF was considered if the patient met at least one organ-dependent criterion and all the general criteria. Vital status of patients at 6 months follow-up was verified by the researchers of each hospital. For this purpose, local health databases were used or relatives were contacted (Supplementary File S1).

### 2.3. Statistical Analysis

Continuous variables were expressed as mean (standard deviation) or median (with 25th to 75th interquartile range), and categorical variables as frequencies and percentages. Continuous variables were compared using Student’s *t*-test or non-parametric Kruskal–Wallis test. Categorical variables were compared using the Chi-square test.

Both groups (HF with and without COPD) were compared. A Kaplan–Meier survival analysis was performed to evaluate the impact of the presence of COPD on the vital prognosis of patients with HF. A *p*-value of less than 0.05 was considered statistically significant. All analyses were performed with the Statistical Package for the Social Sciences (SPSS) program (version 26.0, SPSS Inc., Chicago, IL, USA).

### 2.4. Ethical Aspects

The study was carried out in accordance with the Declaration of Helsinki. Ethical approval (Ethics Committee of the Hospital Virgen Macarena, Internal code 0942-N-15; 24 November 2015) was obtained before recruitment. All patients signed the informed consent at inclusion.

### 2.5. Results

A total of 3100 patients were included of which 812 had COPD. The mean age in the COPD group was 79.29 ± 10.2 years with a predominance of male sex (63%). Mean LVEF was 53.28% ± 15.78 and mean NT-proBNP was 8936.2 pg/mL ± 1047.51. Of the patients, 21.4% were in NYHA functional class III-IV. Patients with COPD had a more advanced NYHA functional class than patients without COPD. No significant statistical differences were observed between groups in relation to LVEF and NT-proBNP levels (Table 1).

Patients with a history of COPD had a higher frequency of diabetes and chronic kidney disease at admission as well as a higher Charlson comorbidity index with statistically significant differences (4.49 ± 1.76 vs. 3.24 ± 1.8; *p* < 0.001) (Table 1).

In relation to the collected symptoms of advanced and terminal disease, in the COPD group, dyspnea and anxiety were more frequently observed (86.2% vs. 75.3%, *p* = 0.001 and 35.4% vs. 31.2%, *p* = 0.043, respectively). No significant statistical differences were observed between groups in relation to chest pain, nausea, insomnia, delirium, and generalized pain (Table 2). In patients with COPD, presentation in the form of acute pulmonary edema was more frequent than in patients without COPD (21% vs. 14%, *p* = 0.0001). Patients with COPD more frequently experience advanced HF (28.9% vs. 19.4%; *p* < 0.001).

Table 3 shows the treatment received during admission in both groups of patients. Subjects with a history of COPD were more frequently treated with noninvasive mechanical ventilation (7.6 vs. 4.6; *p* = 0.004) and high flow oxygen (14% vs. 11.4%; *p* = 0.018). No statistically significant differences were found between groups in the administration of furosemide doses, use of amines, and oral and subcutaneous morphine. Consultation with the hospital palliative care service during admission was more frequent when patients with HF presented with associated COPD (49/520; 94% vs. 99/1450; 6.8%; *p* = 0.036).

In-hospital and 6-month follow-up mortality was 36.5% (270/740) in patients with COPD vs. 30.7% (639/2080) without COPD (*p* = 0.005). The mean number of hospital admissions during follow-up was higher in patients with HF and COPD than in those with isolated HF (0.63 ± 0.98 vs. 0.51 ± 0.84; *p* < 0.002).

**Figure 1:**
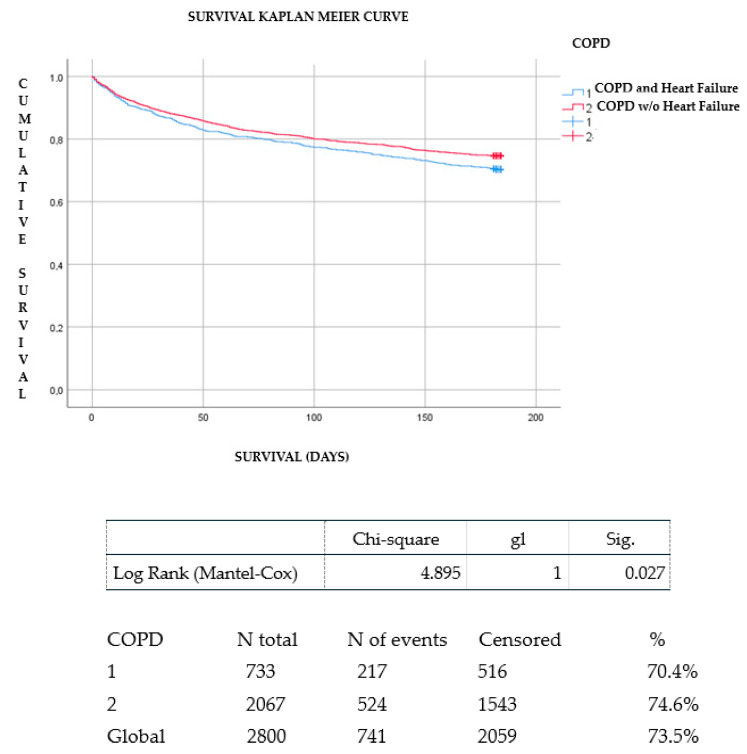
Kaplan–Meier survival curve between HF patients with and without COPD.

Survival analysis showed that patients with a history of COPD had fewer survival days during follow-up than those without COPD (log Rank chi-squared 4.895 and *p* = 0.027). (Figure 1). The causes among patients with acute HF with and without COPD are shown in Supplementary Table S1.

## 3. Discussion

The results of our research demonstrate that six month mortality after hospital admission in elderly acute HF patients was higher in those subjects with a medical antecedent of COPD. This entity confers an even greater risk of dying following hospitalization for acute HF as reported in previous studies [9]. Measures of the severity of COPD (FEV_1_ and GOLD [Global Initiative for Chronic Obstructive Lung Disease] stage) have been shown to be independent predictors of mortality and event-free survival, respectively, in patients with COPD and concomitant HF [11]. In addition, the impact of COPD on mortality in acute HF seems to be more important over the long term [9]. In the OPTIMIZE-HF registry of patients hospitalized with acute HF there were no differences in in-hospital or 60-day mortality rates between patients with and without COPD [12]. However, in our study, we observed a high mortality rate of more than 30% at six months of follow-up, which was even higher for patients with COPD. The differences in the short-term prognosis observed in the different published studies show that our sample of patients included in the EPICTER registry with HF and COPD were in a more advanced and terminal phase of the disease [12].

The presence of COPD was more frequent in the men in our sample. This finding may be due to a higher frequency of smoking in elderly patients admitted for HF in our environment [13].

The presence of COPD was also accompanied by a greater number of comorbidities associated with HF such as diabetes and the presence of previous chronic kidney disease. These findings may be because COPD is also associated with an increased risk of diabetes, among which the use of steroids to control flare-ups may contribute to worsened glycemic control in this type of patient. It is well known that diabetes, previous smoking, and advanced age contribute to the development of chronic kidney disease [1,2,3].

Patients with HF and COPD in the EPICTER study presented more symptoms of advanced HF reporting more dyspnea, anxiety, and need for more use of high oxygen mask supply and noninvasive mechanical ventilation than patients with isolated HF. These findings may be explained by the presence of both conditions in the same patients which imparts a negative impact upon them.

It is very common that HF produces disorders in pulmonary ventilation and perfusion that aggravate COPD and, conversely, that COPD itself, through hypoxemia and/or hypercapnia, increases the risk of cardiac arrhythmias and worsens pulmonary congestion. These findings could explain why COPD patients presented more frequently in acute pulmonary edema as the HF presentation in this study. The findings of our investigation show that the palliative care team evaluated patients with HF and COPD more frequently. There was no difference in the palliative treatment received. Interestingly, although HF is generally considered as a serious condition and equivalent to malignant disease in terms of symptom burden and mortality, only a few patients receive specialist palliative care [14,15,16]. However, evidence indicates that a palliative approach in HF significantly improves patient outcomes, including symptom control and mental health, decreased hospital admissions and mortality, and reduced healthcare costs [17,18].

Interestingly, there were no differences in the use of levosimendan in both groups. Levosimendan augments the calcium sensitivity of the troponin complex subsequently improving cardiac muscle contractility [19,20]. Besides, it has also been shown to improve contractility of the diaphragm [19]. In advanced HF patients, levosimendan has showed positive effects in reducing mortality and three months hospitalization [21,22]. Regarding quality of life and symptoms improvement levosimendan showed contrasting effect among the studies on advanced HF patients [21,23].

The study has some limitations. First, we must consider that the COPD variable was established as an antecedent in the EPICTER study data collection. In this regard, we do not know if the diagnosis of COPD was based on spirometric criteria or on concomitant treatment received for COPD. Second, in-hospital mortality was not differentiated from that of patients surviving admission. Since this was an acute HF registry, only drugs for the control and treatment of acute HF were collected. We do not have specific treatments for chronic HF or COPD. We did not evaluate data regarding congestion such as the presence of pleural effusion as it has related to more cardio-respiratory complications with higher mortality [22]. Finally, not all centers had the same access to specialized palliative care. These aspects are important when interpreting our results. A strength of the study is that the large cohort is prospective and unselected, so it is representative of the real world.

## 4. Conclusions

Patients with HF and COPD had more severe symptoms (dyspnea and anxiety) and also a worse prognosis than patients without COPD. However, the prognosis of patients admitted to our setting is poor and many patients with HF and COPD may not receive the assessment and palliative care support they need. Palliative care is necessary in chronic non-oncologic diseases, especially in multipathologic and symptom-intensive patients. This is a clinical care aspect to be improved and evaluated in future research studies.

## Figures and Tables

**Table 1:** Clinical characteristics of patients with HF with/without history of COPD.

Variable	HF with COPD (n = 812)	HF without COPD (n = 2288)	*p*
Age (years) mean ± SD	77.5 ± 10.1	79.4 ± 11.1	0.001
Sex (male), N (%)	575 (70.8%)	955 (41.7%)	0.001
NYHA III-IV, N (%)	233/802 (29.1%)	486/2241 (21.7%)	0.001
LVEF, mean ± SD	50.08 ± 16.5	51.26 ± 15.9	0.65
NTpro-BNP pg/mL, mean	7910.4	8550.2	0.4
Comorbidities			
Charlson comorbidity index mean ± SD	4.49 ± 1.76	3.24 ± 1.8	0.0001
Hypertension N (%)	694/809 (85.8%)	1936/2283 (84.8%)	0.528
Diabetes N (%)	397/810 (49%)	1010/2279 (44.3%)	0.022
Ischemic heart disease N (%)	271/804 (33.7%)	713/2257 (31.6%)	0.271
Atrial fibrillation N (%)	470/809 (58.1%)	1286/2281 (56.4%)	0.409
Valve disease N (%)	176/377 (37.8%)	1012/2194 (46.1%)	0.001
Chronic kidney disease N (%)	410/807 (50.8%)	1041/2270 (45.9%)	0.017
Cerebrovascular disease N (%)	164/797 (20.6%)	498/2269 (21.9%)	0.453
Anemia N (%)	382/806 (47.4%)	1118/2274 (48.7%)	0.390

Legend: NYHA: New York Heart Association functional class, LVEF: left ventricular ejection fraction, COPD: chronic obstructive pulmonary disease. The diagnosis of acute HF was based on 2016 ESC clinical practice guidelines. COPD was considered if the patient presented the diagnosis of the disease according to their medical history. The diagnosis of anemia was established according to the definition of the World Health Organization (<13 g/L hemoglobin for men and <12 g/L hemoglobin for women). Chronic renal failure was defined as a persistent glomerular filtration rate below 60 mL/min/according to MDRD at least three months.

**Table 2:** Clinical manifestations of advanced disease evaluated among elderly acute HF patients with and without COPD.

Variable	HF with COPD n = 528	HF without COPD n = 1480	*p*-Value
Dyspnea N (%)	455/528 (86.2%)	1114/1480 (75.3%)	0.0001
>10% Weight loss N (%)	48/356 (13.5%)	121/900 (11.9%)	0.236
Functional impairment N (%)	146/379 (38.5%)	390/715 (35.3%)	0.265
Anxiety N (%)	187/528 (35.4%)	462/1480 (31.2%)	0.043
Nausea N (%)	52/528 (9.8%)	176/1480 (11.9%)	0.231
Chest pain N (%)	99/528 (18.8%)	281/1478 (19%)	0.948
Generalized pain N (%)	157/528 (29.7%)	394/1477 (26.7%)	0.098
Delirium N (%)	83/528 (15.7%)	217/1480 (14.7%)	0.570
Insomnia N (%)	197/528 (37.3%)	507/1478 (35%)	0.222

**Table 3:** Treatment received during admission in groups of heart failure patients with and without COPD.

Variable	HF with COPD (n = 812)	HF without COPD (n = 2288)	*p*
Non-invasive mechanical ventilation n (%)	62 (7.6%)	105 (4.6%)	0.004
High Flow oxygen n (%)	74/529 (14%)	229/2014 (11.4%)	0.018
Nitroglicerine iv	69/805(86%)	237/2281(20.4%)	0.222
Hypertonic saline + furosemide n (%)	15/536 (2.8%)	34/1495 (2.3%)	0.297
Furosemide perfusion n (%)	138/812 (17%)	364/2288 (15.9%)	0.770
Use of amines n (%)	50/812 (6.2%)	110/2288 (4.8%)	0.326
Levosimendan n (%)	11/812 (1.4%)	23/2288 (1%)	
Dialysis n (%)	6/536 (1.1%)	17/1493 (1.1%)	1.000
Oral morphine n (%)	151/536 (28.2%)	379/1497 (25.3%)	0.109
Subcutaneous morphine n (%)	74/472 (15.7%)	223/1304 (17.1%)	0.263
Benzodiazepines n (%)	185/535 (34.6%)	496/1496 (33.2%)	0.292

## Data Availability

Data is contained within the article.

## Supplementary Materials

The following supporting information can be downloaded at: https://www.mdpi.com/article/10.3390/jcm11133709/s1, Supplementary File S1. End stage disease criteria. Table S1. Causes of death in patients with HF with and without COPD in the EPICTER registry. Table S2. Logistic regression analysis of mortality evaluating the COPD variable.
