# Supplementary material for: Chronic Obstructive Pulmonary Disease in Elderly Patients with Acute and Advanced Heart Failure: Palliative Care Needs—Analysis of the EPICTER Study
Source: J Clin Med. 2022 Jun 27;11(13):3709. doi: 10.3390/jcm11133709 (PMC9267665; doi:10.3390/jcm11133709)
Supplement: Supplementary file 1 [file jcm-11-03709-s001.zip › jcm-1690701-supplementary.pdf]

**Supplementary file S1**

End-stage disease considerations applied to HF patients included in the EPICTER registry:

Organ-dependent: the presence of persistent baseline NYHA functional class III/IV (not only during admission), left ventricular ejection fraction < 20%, presence of untreatable angina with secondary heart failure, persistent clinical heart failure despite optimal treatment with ACE inhibitors/ARB II, BB, diuretics, ivabradine, contraindication to any of the following procedures (cardiac transplantation, CRT, coronary revascularization, valve replacement, ventricular or mechanical circulatory assistance) and the presence or recurrence of supraventricular and ventricular arrhythmias resistant to medical treatment.

-if the estimated survival prognosis was expected to be ≤ 6 months (the physician would not be surprised if the patient dies within that period), if the patient/family accept a more palliative than intensive approach of the underlying disease, and if the patient met one of these following criteria:

- Evidence of clinical progression of the disease: based on organ-dependent criteria categories (NYHA, ejection fraction, progression on treatment, etc.).

- Multiple Emergency Department visits or admissions in the last six months.

- Unintentional loss of 10% of weight, decrease in albumin in the last six months.

- Obvious functional impairment (assessment made with the caregiver/family member): Barthel test (dependence on at least three daily life activities), Pfeiffer test (> three errors), the presence of lack of adequate socio-familial support.

- Agreement between several physicians.

**Compliance with end-of-life criteria**: at least one of the organ-dependent criteria and all (the three) general end-of-life criteria must be met.

Supplementary Table S1. Causes of death in patients with HF with and without COPD in the EPICTER registry.

| | Sudden death | Myocardial infarction | HF | Stroke | Sepsis/ infection | Pulmpnary embolism | Cancer |
|---|---|---|---|---|---|---|---|
| COPD and HF | 10 | 4 | 102 | 4 | 45 | 1 | 17 |
| HF without COPD (n) | 30 | 15 | 267 | 13 | 99 | 1 | 24 |

Supplementary Table S2. Logistic regression analysis of mortality evaluating the COPD variable.

| | OR | Confidence interval | P |
|---|---|---|---|
| Age | 0.95 | 0.94-0.96 | 0,000 |
| Sex | 1.06 | 0.84-1.34 | 0.603 |
| Left ventricular ejection fraction | 1.01 | 1.007-1.021 | 0.000 |
| COPD | 1,27 | 0.99-1.64 | 0.055 |
